# Measurement of local two-phase flow parameters of nanofluids using conductivity double-sensor probe

**DOI:** 10.1186/1556-276X-6-284

**Published:** 2011-04-04

**Authors:** Yu sun Park, Soon Heung Chang

**Affiliations:** 1 Department of Nuclear and Quantum Engineering, KAIST, 335 Gwahak-ro, Yuseong-gu, Daejeon 305-701, Republic of Korea

## Abstract

A two-phase flow experiment using air and water-based γ-Al_2_O_3 _nanofluid was conducted to observe the basic hydraulic phenomenon of nanofluids. The local two-phase flow parameters were measured with a conductivity double-sensor two-phase void meter. The void fraction, interfacial velocity, interfacial area concentration, and mean bubble diameter were evaluated, and all of those results using the nanofluid were compared with the corresponding results for pure water. The void fraction distribution was flattened in the nanofluid case more than it was in the pure water case. The higher interfacial area concentration resulted in a smaller mean bubble diameter in the case of the nanofluid. This was the first attempt to measure the local two-phase flow parameters of nanofluids using a conductivity double-sensor two-phase void meter. Throughout this experimental study, the differences in the internal two-phase flow structure of the nanofluid were identified. In addition, the heat transfer enhancement of the nanofluid can be resulted from the increase of the interfacial area concentration which means the available area of the heat and mass transfer.

## Introduction

The conventional method of increasing the cooling rate is to use extended heat transfer surfaces for exchanging heat with a heat transfer fluid. However, because this approach requires an undesirable increase in the size of the system, there is a need to develop advanced cooling techniques and innovative heat transfer performances than those presently available. Over the last several decades, engineers have attempted to develop fluids which offer better cooling performances for a variety of thermal systems compared to conventional heat transfer fluids. This motivation inspired Choi [1] to pioneer the development of nanofluids. A nanofluid is a new type of fluid that consists of uniformly dispersed and suspended nanometer-sized particles or fibers in fluids with unprecedented thermal characteristics.

Numerous research groups from around the world have published a large number of experimental and theoretical studies on nanofluids. A certain group argued that nanofluids substantially enhance the heat transfer rate compared to the pure water, while the others found that the inclusion of nanoparticles degraded the boiling performance with increasing the particle concentration. Despite these conflicting research results, the impact of nanofluid technology is expected to be great considering that the heat transfer performance of heat exchangers is vital in numerous industries. In addition, due to the small size of nanoparticles and low volume fraction, problems such as sedimentation, clogging, and abrasion become insignificant with the reduction in required pumping power.

While a considerable body of research exists regarding the heat transfer characteristics of nanofluids, the basic hydraulic phenomenon of a nanofluid, especially in the two-phase flow region, has not been investigated as much. Moreover, there was no attempt to identify the internal structure of the two-phase flow of nanofluids. Hence, in this study, a two-phase flow experiment using an air-nanofluid was conducted. To observe the basic hydraulic phenomenon of nanofluids, the local two-phase flow parameters such as void fraction distribution and interfacial area concentration were measured using a conductivity double-sensor two-phase void meter in a vertically upward air-water two-phase flow. The results obtained from the nanofluids were compared with the results obtained from pure water.

## Experimental apparatus

**Figure 1:**
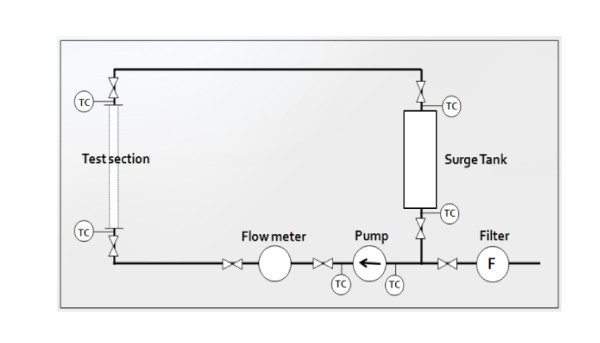
**An overview of the experimental test loop**.

The overall test loop setup is shown in Figure 1. The setup consists of a tank in which the working fluid is stored, a pump circulating the working fluid at a variable speed, and the test section. There are six K-type thermocouples that measure the bulk temperatures of the working fluid. Measured temperatures were used to determine the fluid properties which were required to evaluate the experimental results. The measurement uncertainty of the thermocouples was estimated to be 2.2°C. The volume flow rate of the liquid is measured with a TOSHIBA LF400 flow meter (TOSHIBA Corporation, Tokyo, Japan) at an uncertainty level of about 0.1%. The air flow rate is controlled by an air Viton O-ring mass flow controller, (model M3030V; manufactured by Line Tech 400, Daejeon, Korea). The measurement error rate of the air flow meter is estimated to be less than 1%. The total volume of the test loop is about 288 L, and only 60 L of the working fluid is circulated in the test loop. The working fluids are water, air, and a water-based nanofluid; they are all used under atmospheric pressure.

**Figure 2:**
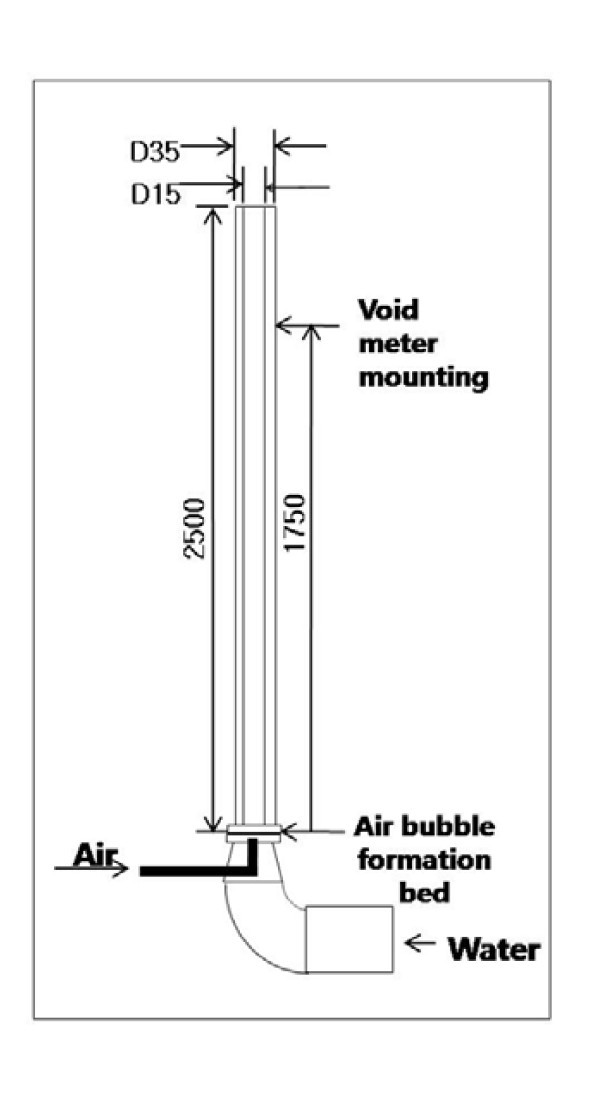
**Specified design of the test section**.

Test section is a vertically oriented acrylic tube as shown in Figure 2. The inner diameter of the test section is 0.015 m and the total height is 2.5 m to ensure that the L/D exceeds 100. Nanofluid and air are mixed at the bottom of the test section and driven by a pump to flow upward. For the bubble formation in the flow, a bubble formation bed is installed on the right before the test section inlet. There are 61 small holes each with a diameter of 1 mm, and they are spaced 2 mm from each other on the bubble formation bed.

In this experiment, a double-sensor two-phase void meter was used as the phase identifier for the two-phase mixture. The conductivity double-sensor two-phase void meter was first proposed by Neal and Bankoff [2]. The double-sensor electrodes consist of two exposed tips, a front sensor and a rear sensor, besides an electrically insulated metal wire and work independently. By considering the fundamental difference in the conductivity between water and air, the circuit is closed when the sensor is in the liquid and is opened when the sensor is in contact with air. The voltage drop across the sensor fluctuates between two reference voltages when the circuit is opened and closed. The information recorded from each signal includes the number of bubbles that strike the sensor, the time that the sensor is exposed to the gas phase, the relative time between the bubble striking the front and rear sensor, and the total sampling time. This information is used to calculate the local two-phase flow parameters: namely, the void fraction, the bubble diameter, the interfacial velocity, and the interfacial area concentration.

**Figure 3:**
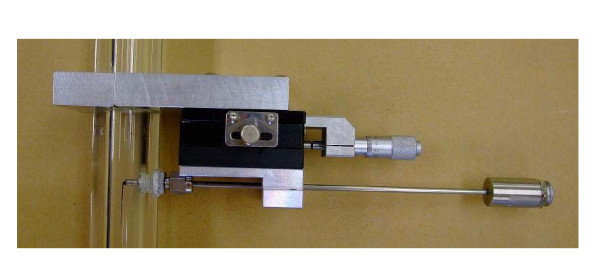
**Mounting the conductivity double-sensor two-phase void meter on the test section**.

The conductivity double-sensor two-phase void meter is mounted at a height of 1.75 m from the bottom of the test section as shown in the Figure 3. The position of the L-shape sensor tip in the radial direction is controlled by a micrometer attached onto the sensor. The output voltage of two-phase identification signal is obtained for 2 s at a 50-kHz sampling frequency. Three times of measurement were conducted at a total of 15 points from the center to the tube inner wall, and the averaged value at each point was used for the analysis. In this study, the same type of a conductivity double-sensor two-phase void meter which was used by Walter [3] was installed and the measurement uncertainty of the void meter is estimated to have a maximum value of 10.5%.

In this study, the bubbly flow regime and the slug flow regime were investigated. The flow regime map proposed by Mishima and Ishii [4] was used to identify each flow regime. As shown in Table 1, a total of 13 flow conditions for the bubbly and slug flow regimes were selected with proper superficial velocities.

**Table 1:** Test cases for the local two-phase flow measurement

Case number	**Liquid flow rate (m**^**3**^**/s)**	**Air flow rate (m**^**3**^**/s)**	Flow regime	Case number	**Liquid flow rate (m**^**3**^**/s)**	**Air flow rate (m**^**3**^**/s)**	Flow regime
1	0.00026	0.000033	Bubbly	8	0.0006	0.000083	Bubbly
2	0.00039	0.000513	Slug	9	0.0005	0.00005	Bubbly
3	0.00039	0.000890	Slug	10	0.0005	0.000033	Bubbly
4	0.00055	0.000513	Slug	11	0.00018	0.000513	Slug
5	0.00055	0.000890	Slug	12	0.00018	0.000033	Bubbly
6	0.00056	0.000513	Slug	13	0.00018	0.000333	Slug
7	0.00056	0.000033	Bubbly	-			

For the synthesis of nanofluid, γ-Al_2_O_3 _nanoparticle powder manufactured by Nanostructured & Amorphous Materials Inc. (Houston, TX, USA) was used. The average particle size of the powder was 25 nm at 99.97% purity based on the information provided by the manufacturer. After the mixing of the γ-Al_2_O_3 _powder with distilled water, it was placed in an ultrasonic bath for an hour for particle dispersion. The nanofluid was then placed in a room temperature atmosphere for 24 h to form an electrical double layer, which makes the nanofluid more stable. This synthesized nanofluid was placed in the ultrasonic bath again for 1 h immediately before the experiment. For a stability check, the zeta potentials were measured before and after the experiments for several concentrations of the γ-Al_2_O_3 _nanofluid. The average values are shown in Table 2; the most stable case of 0.1% was the target concentration for the analysis and discussion.

**Table 2:** Zeta potentials and particle sizes of the synthesized nanofluids

**Volume percent of γ-Al**_**2**_**O**_**3**_	Zeta potential (mV)		Particle size (nm)	
	Before	After	Before	After
0.01	31.93	26.27	100.13	169.48
0.1	42.33	36.88	158.43	142.73
1	-	-	125.15	133.15

## Data reduction

### Fluid properties

The physical properties of the density and viscosity of the nanofluid were calculated using the published correlations shown below. The density of the nanofluid was calculated with the following equation from Pak and Cho [5]:(1)

The viscosity of the nanofluid was obtained from Equation 2 which was suggested by Drew and Passman [6].(2)

Equation 2 can be applied to volume fractions of less than 5.0 vol.%. In the present study, the volume concentration of nanoparticle used was 0.1%; thus, this equation can be applied to estimate the viscosity of the nanofluid [7].

### Void fraction

In general, the area-averaged gas fraction is referred to as the void fraction. If the cross-sectional area of the channel is *A *and the cross-sectional areas occupied by the gas and liquid phases are *A*_g _and *A*_f_, respectively, then the void fraction is given by(3)

In this experiment, the time-averaged void fraction, *α*, is evaluated as a function of the total sampling time, *Ω*, and the total collected pulse widths of the front sensor during the sampling period [3]. The bubble residence time *t*_F1 _- *t*_F2 _is required. It is calculated by Equation 4(4)

### Interfacial velocity

The interfacial velocity can be calculated by taking into account the distance between the tips of the front and rear sensor, Δ*s*, and the time difference between the front and rear signal, *t*_F1 _- *t*_R1 _[3]. The distance between the tips of the front and rear sensor of the conductivity double-sensor two-phase void meter which was used in this experiment was 1.229 mm. The time-averaged interfacial velocity is determined by Equation 5.(5)

### Interfacial area concentration

The interfacial area describes the available area for the interfacial transfer of the mass, momentum, and energy. The interfacial area concentration is defined as the interfacial area per unit volume of the mixture. Its mathematical formula was proposed by Ishii [8].

Measurements of the directional cosines of the sensor and the three-dimensional components of the velocity vectors are used as follows to calculate the time-averaged interfacial area concentration:(6)

Here,  and *φ*_*j *_are the interfacial velocity of the *j*th interface and the angle between  and the unit normal vector of the *j*th interface, respectively [3].

### Sauter mean diameter

The droplet size distribution is frequently characterized by the Sauter mean diameter (a term originally developed by Sauter, a German scientist, in the late 1920s). The Sauter mean diameter is the diameter of a sphere that has the same volume to surface area ratio as a particle of interest. It is typically defined in terms of the surface diameter, *d*_s_, and the volume diameter, *d*_v_. The surface diameter is expressed as(7)

And the volume diameter is expressed as(8)

where *A*_p _and *V*_p _are the surface area and volume of the particle, respectively. The Sauter mean diameter for a given particle can then be expressed as(9)

In this study, the Sauter mean diameter is obtained from the time-averaged interfacial area concentration and the void fraction. That is,(10)

## Results

**Figure 4:**
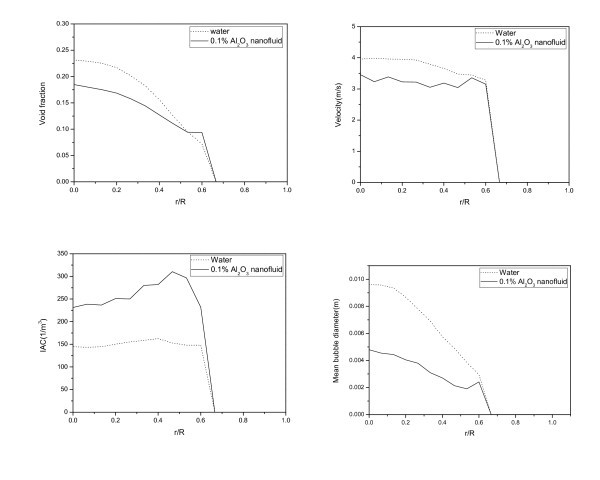
**Comparison of the local two-phase flow parameters in the bubbly flow regime**. Between the pure water and the nanofluid in the bubbly flow regime (*j*_f _= 2.8294 m/s, *j*_g _= 0.1886 m/s).

**Figure 5:**
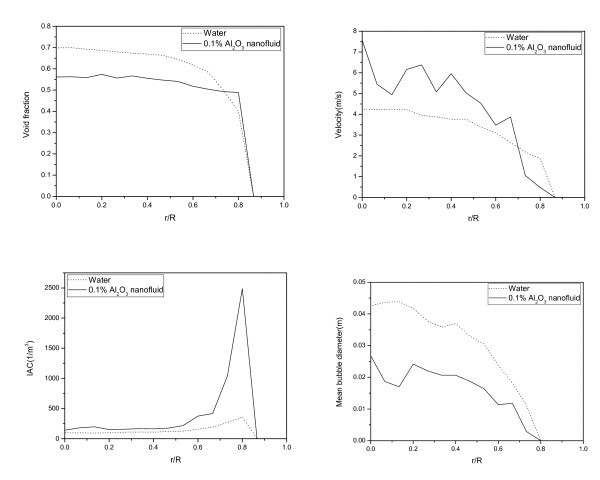
**Comparison of local two-phase flow parameters in the slug flow regime**. Between the pure water and the nanofluid in the slug flow regime (*j*_f _= 1.0186 m/s, *j*_g _= 2.9049 m/s).

The local two-phase flow parameters such as the void fraction, the velocity, the interfacial area concentration, and the bubble diameter were evaluated in the bubbly and slug flow regimes. The results are shown in Figures 4 and 5.

In the bubbly flow regime, as shown in Figure 4, the maximum value of the void fraction distribution is approximately 0.18 in the case of the nanofluid; this value is smaller than that of pure water, 0.225, at the center of the test section. The decrease in the rate of occurrence of void fractions in the nanofluid becomes smaller than that of pure water as the sensor approaches the wall. Thus, the overall shape of the void fraction distribution was flattened more in the case of nanofluids than in the case of pure water. The bubble velocity also decreased in the case of the nanofluid. However, the interfacial area concentration was increased and it was significant as the sensor approached to the wall. And the mean bubble diameter, as determined from the void fraction and interfacial area concentration, was decreased.

In the slug flow regime, as shown in Figure 5, a wider and flatter void fraction distribution compared to that of the pure water was also shown in the nanofluid results. The bubble velocity in the nanofluid case shows a value that is higher than that of the pure water case near the center of the test section. The interfacial area concentration of the nanofluid case also shows a higher value compared to the pure water. Especially in the case of the nanofluid, the interfacial area concentration increased significantly in the vicinity of the wall. This can be concluded that the boundary of air slug and liquid film is located at this point, and the shorter lengths of air slugs pass the void meter in the nanofluid case than in the pure water case. In the mean bubble diameter result, the smaller air slug size in the nanofluid case than that in the pure water case was evaluated as it was reflected in the interfacial area concentration result.

## Discussion

In this experiment, the void fractions were flattened with smaller bubbles in the case of nanofluids. The flattening of the void fraction distribution in the nanofluid can be explained by the forces that act between the two phases. The types of forces that act between the two phases include drag force, lift force, wall lubrication force, and turbulence dispersion force. The main determinant of the transverse motion of the bubbles is the interaction between the drag force and the lift force.

For an evaluation of the drag force, the drag coefficient is derived from the Grace model, which is considered to be an appropriate model for sparsely distributed fluid particles. It is expressed as(11)

**Figure 6:**
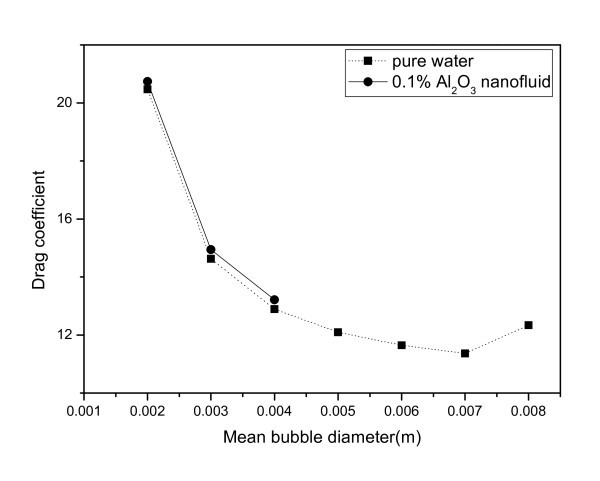
**Drag coefficient in terms of the mean bubble diameter**.

The derivation of the terminal velocity, *U*_T_, is outlined in the ANSYS CFX Solver Theory Guide (ANSYS, Inc., Canonsburg, PA, USA) [9]. To evaluate the drag coefficient using the Grace model, mean bubble diameter is the starting point. As shown in Figure 4, mean bubble diameter ranges from 0 to 0.0079 m for the pure water and from 0 to 0.0034 m for the nanofluid. Within this range of bubble sizes, the drag coefficients are calculated with the fluid properties of the pure water and the nanofluid; the results are shown in Figure 6. The drag coefficient of the small bubbles is about 13 to 22 in the nanofluid and almost 12 in the pure water. In addition, the drag coefficient of the nanofluid is larger than that of the pure water (about 6%) within the same bubble sizes. Thus, the drag force acting on the rising bubbles in the nanofluid case is larger than in the pure water case.

A correlation proposed by Tomiyama [10] was used to evaluate the effect of the lift force. A study of single bubbles in a well-defined shear field was performed by Tomiyama, and the correlation for the lift force coefficient was derived by his experiments:(12)

with(13)

This coefficient depends on the modified Eotvos number, which is given by(14)

The modified Eotvos number can be calculated by using the following empirical correlation of Wellek et al. [11] for the aspect ratio:(15)

**Figure 7:**
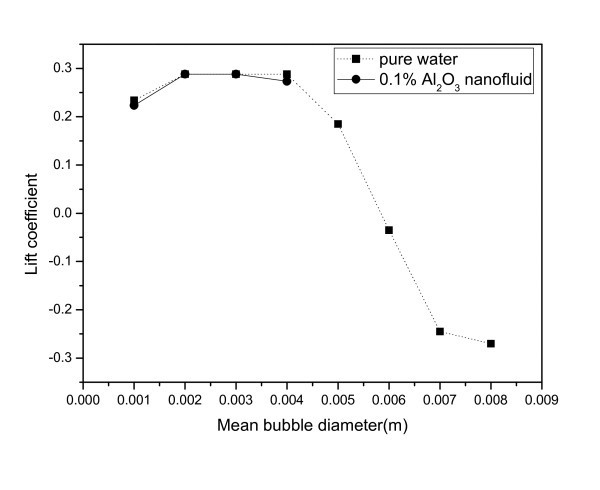
**Lift coefficient in terms of the mean bubble diameter**.

The evaluation results of the lift force are shown in Figure 7. The negative lift coefficients of large bubbles in pure water indicate that the lift force is acting in a direction of the center of the test section. Some large bubbles in the pure water are forced to the center of the test section, and some small bubbles in the pure water are forced to the inner wall of the test section; together they form a void fraction distribution with a center-peaked shape. However, in the nanofluid case, the lift coefficient is always positive, which means that the force acting on the bubbles is in the direction of the inner wall of the test section. Thus, smaller bubbles in the nanofluid shift from the center to the wall, and the void fraction distribution in this case becomes flatter than that of the pure water case.

From these results, it can be concluded that the flattened void fraction in the nanofluid means that the bubbles in the nanofluid smaller than those of pure water were passed in the flow under the force acting in the direction of the wall.

## Conclusion

In this experimental study, a basic hydraulic experiment using a water-based γ-Al_2_O_3 _nanofluid was conducted. Air and the nanofluid were used as working fluids in a vertically upward acrylic tube. The local two-phase flow parameters such as the void fraction, the interfacial velocity, the interfacial area concentration, and the mean bubble diameter were measured using a conductivity double-sensor two-phase void meter in bubbly and slug flow regimes. The void fraction distribution was flattened in the nanofluid case more than it was in the pure water case. The higher interfacial area concentration resulted in a smaller mean bubble diameter in the case of the nanofluid. In view of the forces acting between the two phases, the difference between the nanofluid and pure water can be attributed to the smaller bubbles that form in the nanofluid.

Throughout this experimental study, the characteristics of the internal two-phase flow structure of the nanofluid were specified. In addition, the heat transfer enhancement of nanofluid can be resulted from the increase of the interfacial area concentration which refers to the available area of the mass, momentum, and energy transfer.

### Nomenclature

A cross-sectional area (m^2^)

*a*_i _interfacial area concentration (1/m)

*C*_D _drag coefficient

*D *inner diameter of the test section (m)

*d *diameter of a bubble (m)

*g *gravitational acceleration (m/s^2^)

*j *superficial velocity (m/s)

*L *test section length (m)

*N*_t _total number of bubbles that strike the sensor

Δ*s *distance between the tips of the front and rear sensor (m)

*t*_F1 _time that a bubble starts to hit the front sensor (s)

*t*_F2 _time that a bubble departs from the front sensor (s)

*t*_R1 _time that a bubble start to hit the rear sensor (s)

*Z *height of the test section (m)

*Α *void fraction

*ε *energy dissipation rate per unit mass

*μ *viscosity (N.s/m^2^)

*ν *kinematic viscosity (m^2^/s)

*ρ *density (kg/m^3^)

*σ *surface tension (N/m)

*φ *volume fraction of nanoparticle

*Ω *total sampling time (s)

### Subscripts

f liquid phase

g gas phase

nf nanofluid

pw pure water

p nanoparticle

## Competing interests

The authors declare that they have no competing interests.

## Authors' contributions

YS performed the experiment and data analysis, and drafted the manuscript. SHC conceived of this study and participated in its design and coordination and helped to draft the manuscript. All authors read and approved the final manuscript.
